# Retroperitoneal Liposarcoma Presenting With Malignant Gastric Outlet Obstruction and Acute Pancreatitis: A Case Report

**DOI:** 10.7759/cureus.12775

**Published:** 2021-01-18

**Authors:** Luisa M Recinos, Sonmoon Mohapatra, Aadhithyaraman Santharaman, Neil Patel, Arkady Broder

**Affiliations:** 1 Department of Internal Medicine, Saint Peter’s University Hospital/Rutgers Robert Wood Johnson School of Medicine, New Brunswick, USA; 2 Division of Gastroenterology and Hepatology, Saint Peter’s University Hospital/Rutgers Robert Wood Johnson School of Medicine, New Brunswick, USA; 3 Cell Biology and Neuroscience, Rutgers University, New Brunswick, USA

**Keywords:** dedifferentiated liposarcoma, retroperitoneal liposarcoma, duodenal obstruction, malignant gastric outlet obstruction, duodenal stent, acute pancreatitis

## Abstract

Dedifferentiated liposarcomas (DDLPS) are rare, high-grade malignancies that usually originate in the retroperitoneum. Frequently, they present as asymptomatic masses, abdominal distention, abdominal pain, and weight loss. They tend to grow significantly and are usually large in size at the time of diagnosis. Surgical resection is the mainstay of treatment; however, local recurrence is common. When unresectable, they can invade local structures and produce a significant mass effect on the adjacent organs. Here we present the first case of malignant gastric outlet obstruction (MGOO) and acute pancreatitis from a retroperitoneal DDLPS.

## Introduction

Soft tissue sarcomas are rare and heterogeneous group of connective tissue tumors. They originate from mesenchymal tissue and subdivide into more than a dozen subtypes; the most frequent subtypes are leiomyosarcoma and liposarcoma [1]. Liposarcomas are classified into five categories according to the World Health Organization: well-differentiated, dedifferentiated, myxoid, round cell, and pleomorphic.

Retroperitoneal liposarcomas are challenging to treat. Surgery continues to be the only potentially curative approach, but success rates are limited. Locoregional recurrence of retroperitoneal sarcomas is the major, problematic aspect of the disease’s management. Large tumor size at diagnosis is associated with an increased risk of recurrence and death [1,2]. We report a case of dedifferentiated liposarcoma (DDLPS) who presented with two very unusual complications associated with its recurrence.

## Case presentation

A 43-year-old male with a known history of retroperitoneal DDLPS presented to the emergency department complaining of upper abdominal pain with band-like radiation to the back. Associated, he had multiple episodes of vomiting for one day. On physical exam, he was found to have diffuse abdominal tenderness and distention. 

**Figure 1:**
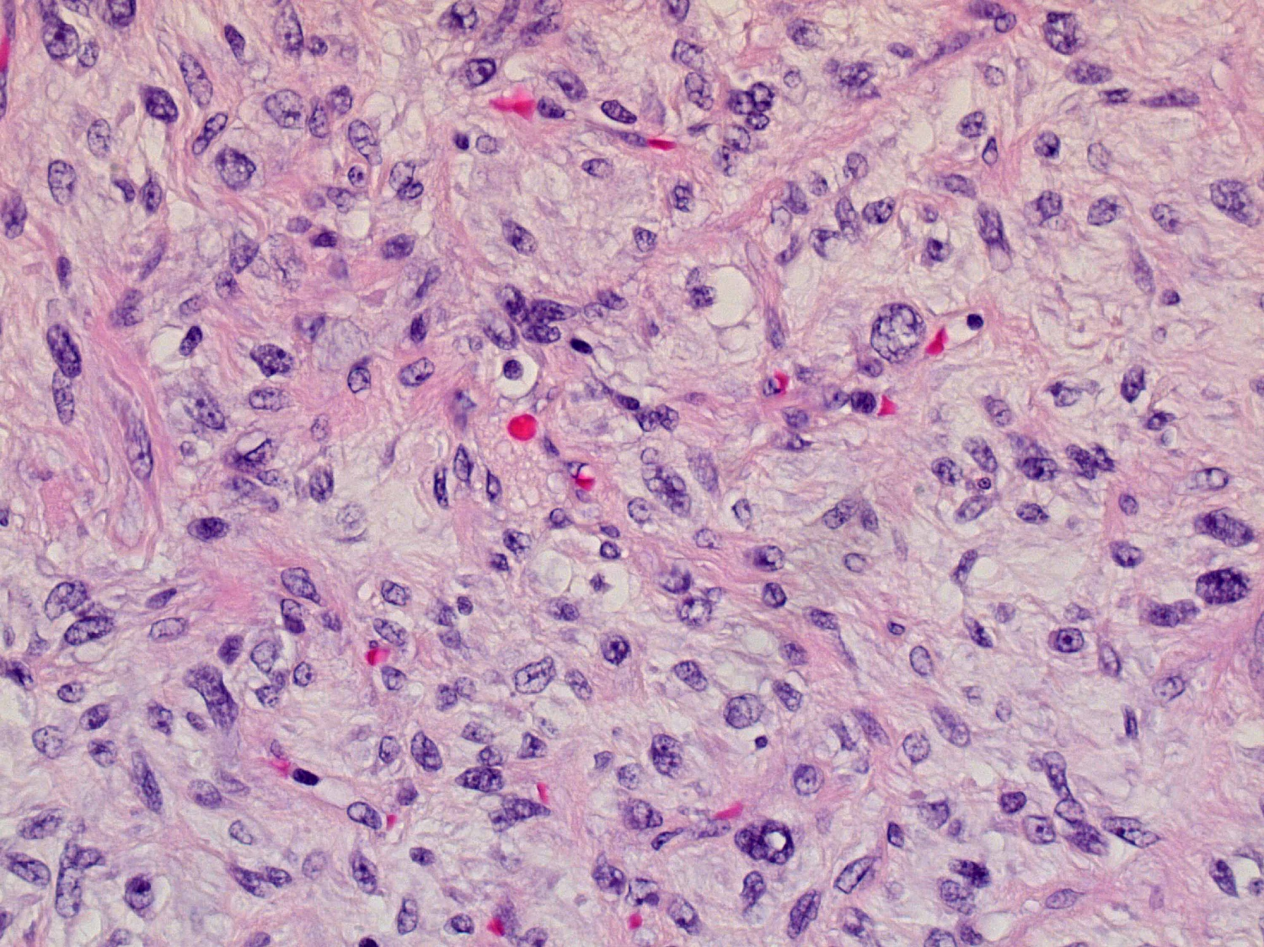
Resected mass histology. Microscopic evaluation (40X magnification): Dedifferentiated liposarcoma.

**Figure 2:**
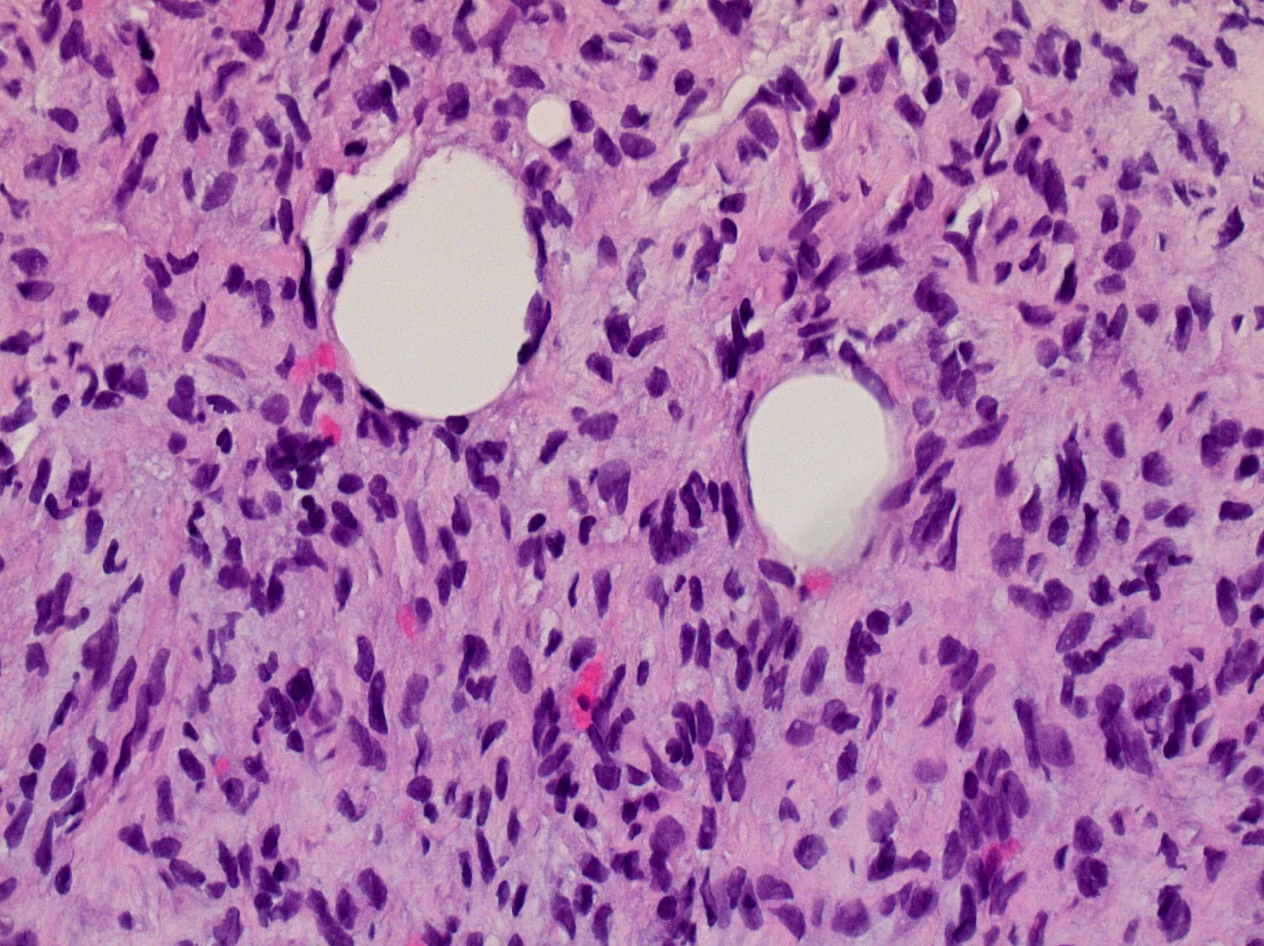
Lymph node biopsy. Microscopic evaluation (40X magnification): Spindle tumor with similar morphology as the original tumor.

The patient had undergone exploratory laparoscopy for tumor resection, and right radical nephrectomy for stage IV retroperitoneal dedifferentiated liposarcoma one year before the presentation. The resected mass was 20 cm in size. The surgical and pathologic margins of the resected specimen were negative at that time. The microscopic findings revealed a spindle mass with fibrous stroma consistent with a Grade 3 dedifferentiated liposarcoma (Figure 1). After six months of resection, lymph node recurrence was noted on surveillance imaging. A lymph node biopsy at that time showed spindle cell sarcoma with similar morphology to the original tumor (Figure 2).

**Figure 3:**
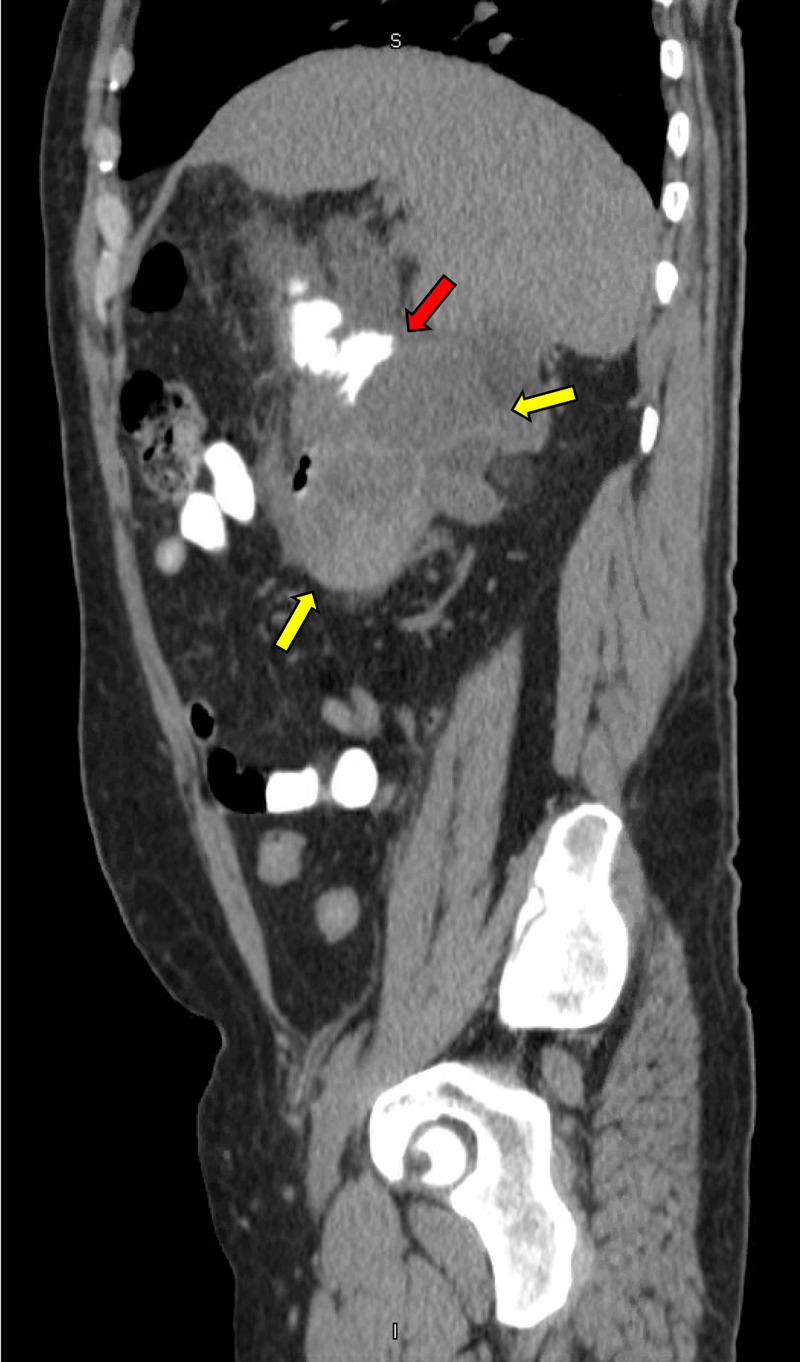
CT scan of the abdomen and pelvis sagittal plane. Right retroperitoneal lobulated masses (yellow arrows) provoking duodenal obstruction (red arrow).

**Figure 4:**
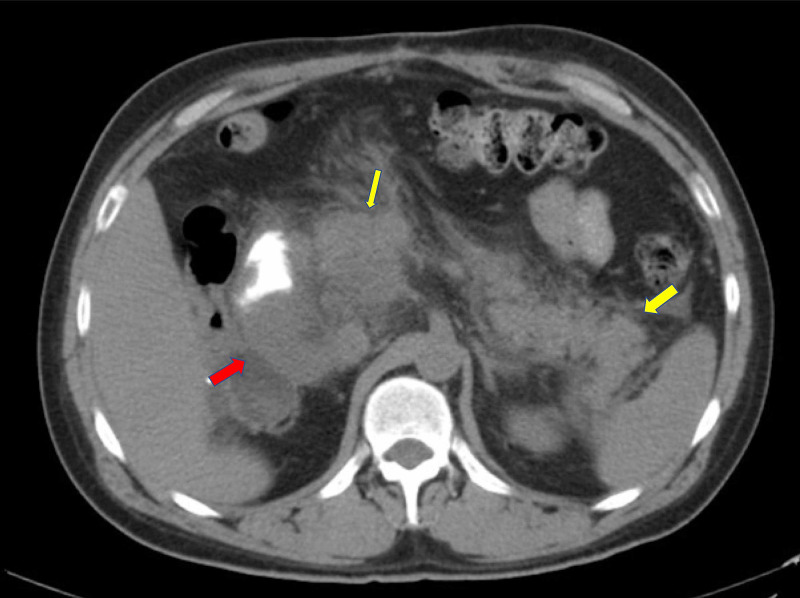
CT scan of the abdomen and pelvis transverse plane. Right retroperitoneal mass (red arrow). Acute pancreatitis with stranding involving the head of the pancreas (yellow thin arrow) and tail (yellow thick arrow). Pancreatic ducts are not visualized.

On admission, a CT scan of the abdomen revealed enlargement of the previously noted masses. The tumor was reported to be 17 cm in size (identified by yellow arrows), extending into the duodenal lumen, causing a duodenal obstruction (Figure 3). The CT scan also revealed acute pancreatitis with significant stranding and free fluid surrounding the pancreas (Figure 4). Serum lipase was 3817 U/L at the time of presentation. 

**Figure 5:**
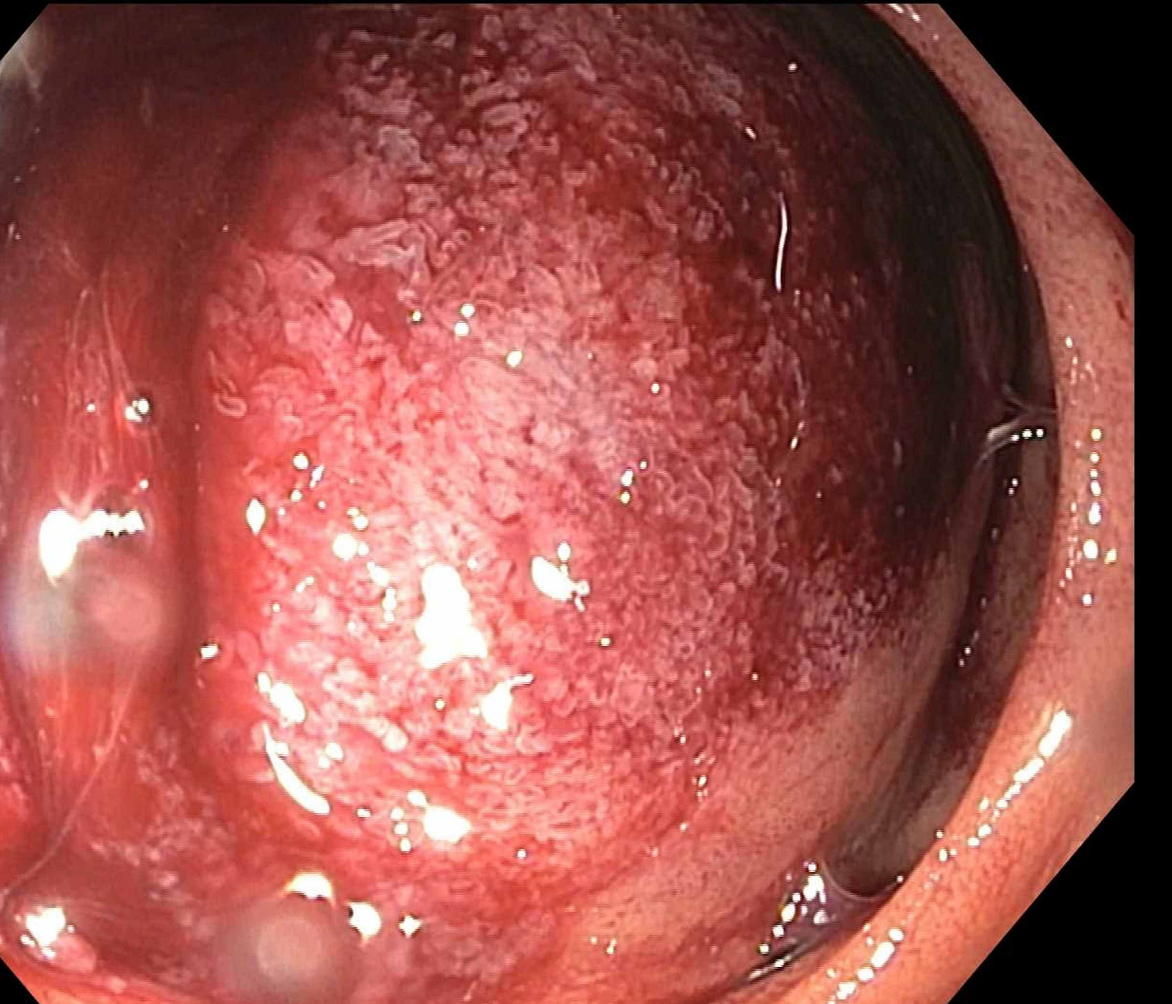
Endoscopic findings Visualization of polypoid duodenal mass obstructing the duodenal lumen.

**Figure 6:**
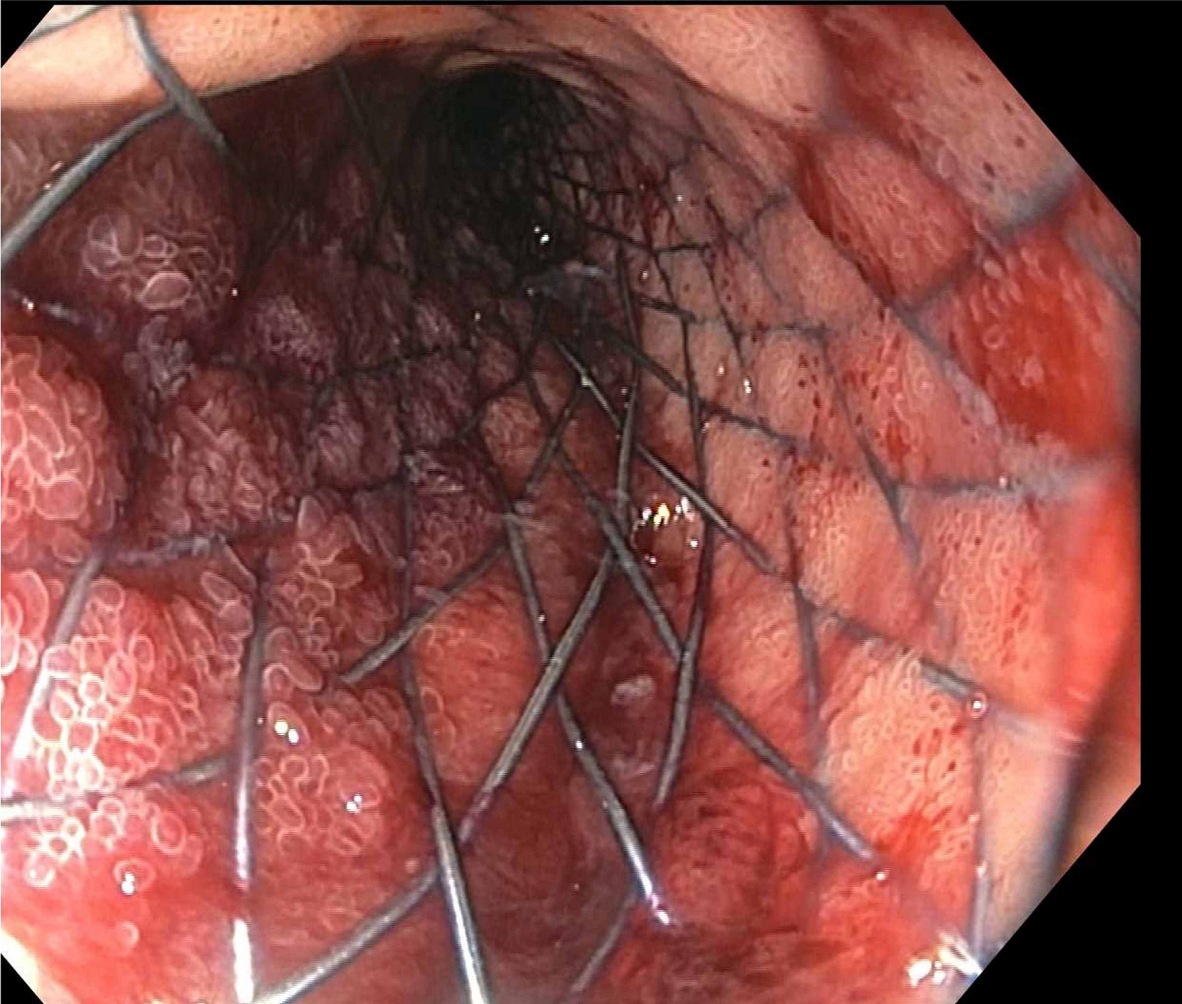
Duodenal stent. Metallic duodenal stent size 22 mm X 12 cm in situ.

**Figure 7:**
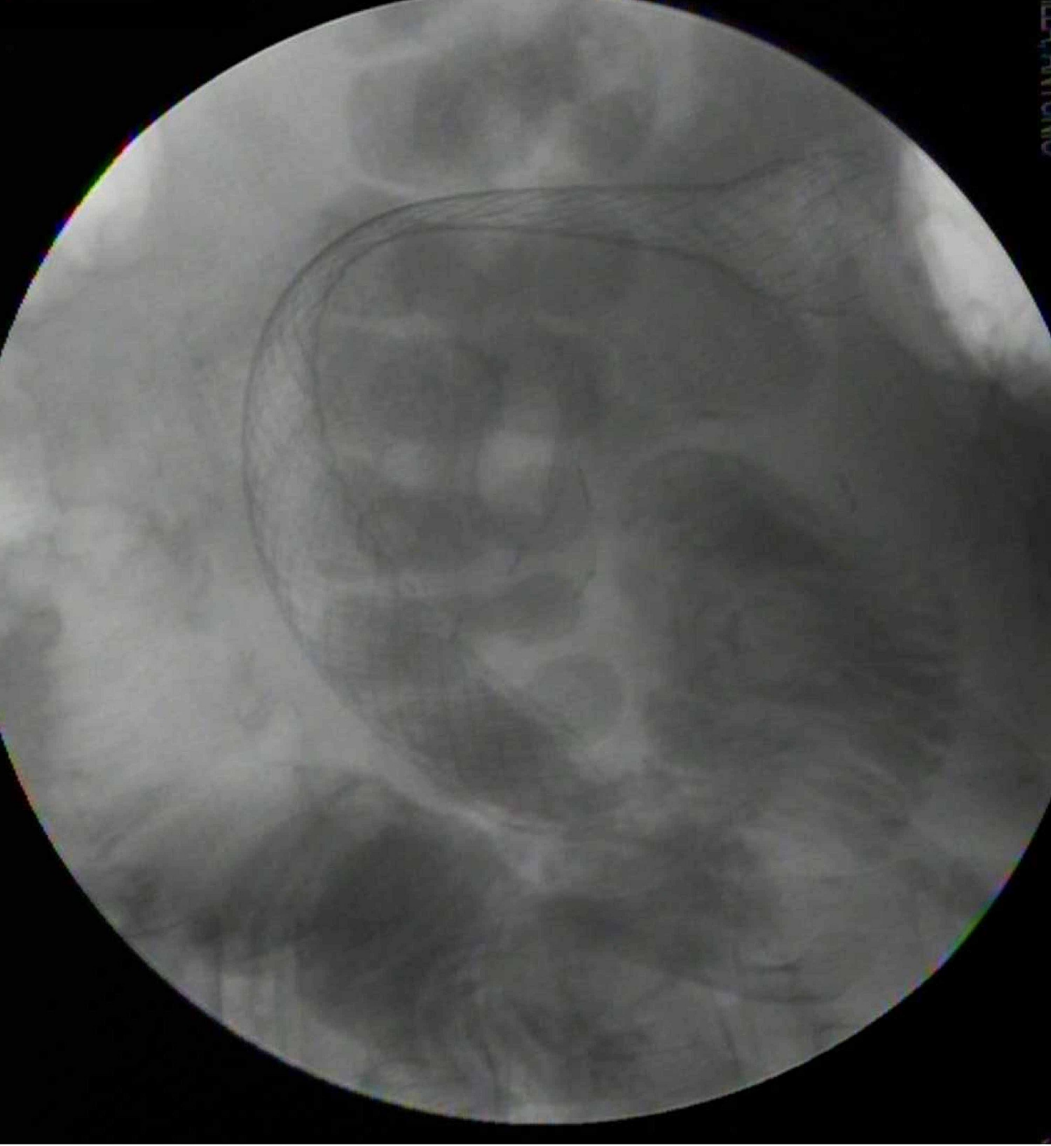
Duodenal stent. Fluoroscopic image of duodenal stent in situ.

Subsequent esophagogastroduodenoscopy demonstrated a large polypoid mass in the second portion of the duodenum, completely obstructing the duodenal lumen (Figure 5). A 22 mm x 12 cm metallic duodenal stent was placed under fluoroscopic guidance (Figures 6, 7). After the duodenal stent placement, his symptoms were significantly improved. After discharge, radiation therapy was attempted which he did not tolerate well. He was also started on doxorubicin and ifosfamide which he did not tolerate due to neurologic side effects. Later, he was treated with pazopanib (a multikinase angiogenesis inhibitor) with evidence of radiographic and symptomatic improvement. 

## Discussion

Retroperitoneal sarcomas are rare tumors with an overall incidence of 0.3-0.4 per 100,000 population [3]. The majority of these tumors are liposarcomas (40%). Well-differentiated liposarcomas (WDLPS) and dedifferentiated liposarcomas (DDLPS) compose the largest subgroups. Both WDLPS and DDLPS are associated with high-level amplifications in the chromosomal 12q13-15 region; however, DDLPS has higher rates of recurrence [4]. DDLPS typically presents in the sixth to eighth decade of life without any gender predilection. Often, they are found as asymptomatic masses on physical exam or detected incidentally on imaging studies. When symptomatic, they tend to present with abdominal pain, abdominal distention, and/or early satiety [5]. At the time of diagnosis, the median tumor size has been reported to be >15cm [2]. 

Malignant gastric outlet obstruction (MGOO) is commonly seen in patients with pancreatobiliary and gastric cancers [6,7] but is extremely rare in cases of retroperitoneal liposarcoma. Acute pancreatitis from retroperitoneal DDLPS has been reported in one previous case [8]. The mechanism of acute pancreatitis in our patient is likely secondary to the compression of the retroperitoneal DDLPS on the second portion of the duodenum and ampulla of Vater, limiting the flow of the pancreatic secretions. Acute pancreatitis is commonly caused by gallstone disease, alcoholism, and elevated triglycerides; none of the above were seen in our patient. 

Other reported complications of retroperitoneal liposarcoma include lumbar or pelvic nerve plexus compression, non-malignant serous ascites, and very rarely, paraneoplastic syndromes (production of granulocyte-colony stimulating factor, alpha-fetoprotein, leukocytosis, pemphigus, and acrokeratosis) [9]. 

Management of DDLPS is typically radical surgical resection with pre-operative chemoradiation for local control [10]. Relative resistance to conventional chemotherapy has been reported [11]. DDLPS local recurrence rate has been estimated to be up to 40%-57% [12], and a mortality rate of 28% [4]. They frequently recur within six months to two years after the initial surgical resection. Even after achieving complete resection and obtaining negative microscopic margins, high local recurrence rates have been reported, probably due to other factors like tumor nature and grade [2]. It has been proposed that, unless extensive sampling is done, inaccuracies may be present in the pathologic margin reports (due to the large size of these tumors and aggressive invasion of adjacent structures) [13]. A short interval CT follow-up (every three months) has been proposed [14]. Endoscopic duodenal stenting can be offered as a palliative measure to relieve the MGOO [6]. 

## Conclusions

A retroperitoneal DDLPS presenting with MGOO and acute pancreatitis is rare, and to the best of our knowledge, has never been reported in the literature. Retroperitoneal DDLPS are highly invasive tumors with high recurrence rates. Thus, close follow-ups with imaging such as CT scans should be considered for monitoring of recurrent disease even after complete surgical excision.
